# Failing Sight

**Published:** 1880-01

**Authors:** 


### FAILING SIGHT.

It is really astonishing how few people attach any importance to their failing vision. Most persons, when they discover that vision is becoming dim, regard it as a natural consequence of approaching age, and complacently wait until they dare brave public inspection with glasses mounted before their eyes. If all cases of failing vision could be attributed solely to changes in the lens structure, to be corrected by spectacles, this general apathy about consulting an oculist and ascertaining the real cause, would not be attended by any harmful consequences. But it so often happens that serious lesions in the nerve, or retina, or both, occur, in which the hapless victim is doomed to incurable blindness, simply because of neglect in obtaining suitable treatment for combating the threatened danger, that we are urged to call the attention of our readers to it.

The painless and almost imperceptible progress of certain diseased conditions of the interior structure of the eye, renders it of the utmost consequence that the nature of the malady should be early recognized and proper remedies applied. Such ailments are too often classed with the ordinary causes of the failure of vision,—such as are found in near and far sightedness, when the patient comforts himself with the reflection that he will apply to a spectacle vender and adjust a pair of spectacles to his eyes, and so immediately overcome his defect in vision.

These reflections are brought about through an interview just held with a gentleman from a neighboring town, who applied to us for aid in restoring his vision. His story is one so often heard by the oculist that it is worth recording as a warning to others likely to fall into the same error.

About four months ago, he first experienced a slight fogginess, or dimness of vision, which lasted but for a few minutes at a time. In a short time after he first noticed this symptom, and which he and his friends attributed to the want of glasses, he would have moments of almost complete blindness, that would speedily pass away and leave only a smokiness of vision when trying to read. Later still, he began to see bright colors about the gas light, which in a few weeks developed into a sort of rainbow over and around the light. But, as he had no pain, and his friends told him his eyes looked bright and natural, he consoled himself with the reflection that it was the beginning of "old sight," and that he would soon put on spectacles and get rid of the annoyance. Finally, one night he was awakened with a pain in his eye balls and temples, but which soon disappeared, and he fell asleep again. In the morning he awoke, and hearing the family astir, and he in total darkness, became alarmed, and called some one to him, when it was discovered that he was completely blind.

His disease is one known to the oculist as *glaucoma*, a curable one in the beginning, but when complete, utterly without means of relief. Had this man applied to any skillful oculist when his symptoms of dimness of vision first manifested themselves, a delicate operation could have been performed (*iridectomy*), by means of which his vision could have been saved. But a heedlessness, or what would be more properly termed a criminal negligence, consigned him to incurable blindness.

We again and again meet with these pitiable cases. Medical journals detail the dangerous symptoms, but these periodicals rarely fall into the hands of the laity, so that they have no warning of their danger. As the BISTOURY is read in the family as well as in the professional study, it is hoped some good may grow out of the timely warnings we are constantly offering.

People are continually committing a great error in attempting to fit their own eyes with glasses. This never should be done. There are so many different forms of defective refraction —to be remedied by spectacles—that cannot be overcome by the trafficer in optical goods, that hundreds of people go plodding through the world without being aware that any remedy exists for their ailment. They have "tried on" glasses at the local spectacle dealers, but failed to find anything to rectify their peculiar trouble. This, then, is to inform all persons so afflicted, that every oculist—and almost every city has one of undoubted skill, now-a-days—has a set of trial glasses, by means of which, and that of a peculiar little instrument that he calls an ophthalmoscope, he can detect every defect in the refractive power of your eyes, and overcome every imperfection of this character, by giving you a prescription to opticians whose business it is to grind such spectacles to order, of any required combination of spheres, cylinders or prisms that is needed.— The spectacles are made to *fit*. The center of the glass should come opposite the center of your pupil, the lens should be round and not oblong. The frames should be steel or bronze and never bright like gold or silver.

Children, when they complain of their eyes at school, or when reading at home, should have an examination made as to the cause of their trouble. They are very liable to oversightedness, which, if neglected, will produce cross-eye. The majority of crossed eyes in school children are produced in an effort to overcome their oversightedness. This should be speedily corrected with suitably fitted spectacles, else will the difficulty progress, and often produce a very serious disease of the interior of the eye, not infrequently resulting in blindness.

